# Design, methodology, and baseline of eastern China student health and wellbeing cohort study

**DOI:** 10.3389/fpubh.2023.1100227

**Published:** 2023-04-27

**Authors:** Xiyan Zhang, Genming Zhao, Jun Ma, Fangbiao Tao, Chen-Wei Pan, Fengyun Zhang, Yan Wang, Wenyi Yang, Yao Xiang, Xin Wang, Yunfan Tian, Jie Yang, Wei Du, Yonglin Zhou, ES-SCI Work Group

**Affiliations:** ^1^Department of Child and Adolescent Health Promotion, Jiangsu Provincial Center for Disease Control and Prevention, Nanjing, China; ^2^School of Public Health, Nanjing Medical University, Nanjing, China; ^3^Department of Epidemiology, School of Public Health, Fudan University, Key Laboratory of Public Health Safety, Ministry of Education, Shanghai, China; ^4^Institute of Child and Adolescent Health, School of Public Health, Peking University, Beijing, China; ^5^Department of Maternal, Child and Adolescent Health, School of Public Health, Anhui Medical University, Hefei, China; ^6^School of Public Health, Medical College of Soochow University, Suzhou, China; ^7^Department of Cardiology, The First Affiliated Hospital of Nanjing Medical University, Nanjing, China; ^8^School of Public Health, Southeast University, Nanjing, China

**Keywords:** cohort, school aged, health, wellbeing, profile

## Abstract

**Purpose:**

To describe the study design, methodology, and cohort profile of the Eastern China Student Health and Wellbeing Cohort Study. The cohort baseline includes (1) targeted disease (myopia, obesity, elevated blood pressure, and mental health) and (2) exposures (individual behaviors, environment, metabolomics, and gene and epigenetics).

**Participants:**

Annual physical examination, questionnaire-based survey, and bio-sampling have been carried out in the study population. In the first stage (2019–2021), a total of 6,506 students in primary schools are enrolled in the cohort study.

**Findings to date:**

Of all the cohort participants, the ratio of male to female is 1.16 among a total of 6,506 student participants, of which 2,728 (41.9%) students are from developed regions and 3,778 (58.1%) students are from developing regions. The initial age of observation is 6–10 years, and they will be observed until they graduate from high school (>18 years of age). (1) Targeted diseases: The growth rates of myopia, obesity, and high blood pressure vary by regions, and for developed regions, the prevalence of myopia, obesity, and elevated blood pressure is 29.2%, 17.4%, and 12.6% in the first year, respectively. For developing regions, the prevalence of myopia, obesity, and elevated blood pressure is 22.3%, 20.7%, and 17.1% in the first year, respectively. The average score of CES-D is 12.9 ± 9.8 in developing regions/11.6 ± 9.0 in developed regions. (2) Exposures: ① The *first aspect of individual behaviors*: the questionnaire topics include diet, physical exercise, bullying, and family. ② *The second aspect of environment and metabolomics:* the average desk illumination is 430.78 (355.84–611.56) L_X_, and the average blackboard illumination is 365.33 (286.83–516.84) L_X_. Metabolomics like bisphenol A in the urine is 0.734 ng/ml. ③ The *third aspect of gene and epigenetics:* SNPs (rs524952, rs524952, rs2969180, rs2908972, rs10880855, rs1939008, rs9928731, rs72621438, rs9939609, rs8050136 and so on) are detected.

**Future plans:**

Eastern China Student Health and Wellbeing Cohort Study is aiming to focus on the development of student-targeted diseases. For children with student common diseases, this study will focus on targeted disease-related indicators. For children without targeted disease, this study aims to explore the longitudinal relationship between exposure factors and outcomes, excluding baseline confounding factors. Exposure factors include three aspects: (1) individual behaviors, (2) environment and metabolomics, and (3) gene and epigenetics. The cohort study will continue until 2035.

## Introduction

The newly established WHO–UNICEF–Lancet Commission has raised the concern of urgent investment in children to achieve the Sustainable Development Goals (SDGs) (1, 2). Previous studies reported that the burden of chronic diseases among children and adolescents is rising remarkably in recent decades in China (3–10). In 2019, the central government released the *Healthy China action (2019–2030)*, with outlines specifically addressing student myopia, obesity, blood pressure, and psychological indicators for evaluation (11).

The Eastern-Chinese Students common disease Surveillance, Cohort, and Intervention study (ES-SCI study) cohort section (**Eastern China Student Health and Wellbeing Cohort Study**) is launched to investigate how the health and wellbeing of students would be impaired or improved *via* the cohort approaches. We focus on targeted diseases concerning student common diseases (myopia, obesity, elevated blood pressure, and mental health). **First, myopia is one of the most commonly observed diseases among eastern Chinese children and adolescents, and the occurrence of myopia in childhood is highly indicative of myopia in adulthood** (12–14). While childhood diseases such as myopia are often irreversible (7), early identification and intervention on modifiable risk factors would potentially reduce the healthcare burden in their adulthood. Therefore, a prospective cohort has been established, similar to previous studies tracking the study population to quantify a range of exposure-outcome associations (15, 16). Meanwhile, the screening and prevention strategies for myopia and the intervention approaches based on follow-up observations of the cohort will also be proposed. A similar school-based cohort study of myopia (cycloplegia) is rare in China. The Tianjin group conducted a non-cycloplegic photo screening study aiming to monitor the progression of refractive error for the same non-cycloplegic population (17). The Anyang Childhood Eye Study is designed to explore the clinical treatment of children’s ophthalmopathy (18). Adding to the literature, the current ES-SCI study focuses on the course of myopia development, taking into account other potentially concurrent common diseases in students. In 2018, the central government issued *an implementation plan for comprehensive prevention and control of myopia among children and adolescents* (19). The document highlights that, by 2030, the incidence rate of myopia among children and adolescents in China should be significantly reduced, as well as the overall level of vision health of children and adolescents should be significantly improved (20). **Second, childhood obesity has become an increasing concern in many countries including China** (21). Located in eastern China, Jiangsu Province is one of the developed regions in China and is also facing this problem. The prevalence of combined overweight and obesity was still maintained at a high level from the previous study in Eastern China. Previously, we found that the prevalence of overweight, obesity, and combined overweight and obesity was 15.6% [95% CI 15.1–16.0%], 11.1% (95% CI 10.7–11.4%), and 26.6% (95% CI 26.1–27.2%), respectively, for female students; and 20.3% (95% CI 19.8–20.8%), 18.8% (95% CI 18.3–19.2%), and 39.1% (95% CI 38.5–39.7%), respectively, for male students. Cohort studies focusing on childhood overweight and obesity should be highlighted in eastern China. **Third, China is one of the world’s largest developing countries with a population of 1.37 billion, and while the prevalence of elevated blood pressure has been stable among Chinese children, this has become an increasing concern recently** (22). Previously, we found that the burden of elevated blood pressure for both female and male students in eastern China was substantially heavy (above the average level among Chinese adolescents). The prevalence of overweight and obesity among primary school children was 15.2% (18.7% for male students and 11.0% for female students) and 11.7% (14.5% for male students and 8.2% for female students), respectively. Potential risk factors can be body status, behaviors and environment. Further cohort study should be taken on (23). **Fourth, *China Youth Development Statistics Report (2020)* shows that about 30 million children and adolescents aged 17 years and under are troubled by various mental health problems**. Primary and secondary school students are in the period of physical and mental development, and their mental sub-health status is particularly noteworthy.

Based on targeted diseases (myopia, obesity, blood pressure, and mental health), this study is designed to observe three major aspects of common diseases in the student participants. Measures for both outcomes and exposures will be carried out at baseline and during the follow-up period for quantifying their associations in children and adolescents. ***The first aspect of individual behaviors and physical growth*:** Physical growth in children and adolescents and corresponding behaviors, such as diet, physical fitness, and psychological status, all have a substantial association with students’ health. Students are vulnerable and deserve special attention on diets because adult health can be affected by nutrition during adolescence (24). Physical growth assessment is a good approach to evaluating students’ health, and perhaps indirectly reflects the socioeconomic status of the family, the social wellbeing of the community, and the efficiency of the healthcare system (25, 26). We are intending to explore the correlation between behaviors and targeted disease-related indicators through long-term observation. ***The second aspect of Environment and metabolomics*:** Environment factors, such as family type/structure, parental education, and the economic level can have an impact on children and adolescents (27–29). Metabolomics could be a powerful approach to childhood health, measuring the exact functioning of individual metabolic responses to environmental changes, diseases, and other epigenetic factors (30). For example, Biphenyl A (BPA) is suspected to be associated with obesity in students, with a 1-ng/ml increase in BPA can increase the risk of obesity by 11% (31, 32). In this study, we are aiming to collect biological samples to explore the relationship between metabolites and targeted diseases and related indicators. ***The third aspect of gene and epigenetics*:** Nature versus nurture has been a frequently debated topic in recent decades. Epigenetics or gene–environment interactions are used to identify candidate biomarkers to assess determinants of common student diseases (33, 34). However, we still know limited about gene–environment interactions in genetic and biological systems concerning students’ common diseases. ES-SCI studies are planning to explore the association between targeted gene–environment interaction-related SNPs and disease.

The overarching aim of **the Eastern China Student Health and Wellbeing Cohort Study** is, first, to describe the fundamental methodology and to summarize the baseline findings for the cohort (targeted diseases such as myopia, obesity, overweight, elevated blood pressure, mental health, and related indicators). Second, to quantify associations between targeted diseases as well as related indicators and three exposure aspects referred earlier and to develop a suite of multidimensional risk assessment tools for diseases commonly seen in children. Third, to provide evidence for intervention strategies to reduce disease burden in students, as well as to improve the health and wellbeing of school-aged children in Jiangsu, China.

## Methods and analysis

### Cohort participants

(1) Sample size calculation

Considering the expected prevalence of targeted disease, expected risk ratio (RR) of exposures from three aspects (individual behaviors, environment and metabolomics, and gene and epigenetics), and students clustered in schools, we assumed parameters for sample size estimation. Based on previous surveillance results in Jiangsu Province in 2018, the expected prevalences of the targeted diseases are as follows: (1) myopia = 18.2%; (2) obesity = 8.15%; (3) elevated blood pressure = 8.0%; and (4) screening mental health (depression) = 8.05%; the expected RRs of three aspects are above 1.5, *α* = 0.05 (significance level of 0.05, *μ_α_* = 1.96), *β* = 0.10 (minimum power of 90%), and design effect is 1.5, allowing for a possible 15% loss to follow-up for reasons such as moving out of the study locations, and therefore, we derived a conservative total sample of 4,154 students from grade 1 to grade 3. We have recruited around 6,506 students for the ES-SCI study eventually.
(2) Inclusion and exclusion criteria: ① Chinese Han Nationality students, aged 6 to 10 years from grades 1 to 3; ② without any other severe diseases, such as hepatitis, nephritis, and eye disease; ③ provision of informed consent from parents/guardians; and ④ school-based participant to prevent loss of visit.

### Study design and area

**Figure 1:**
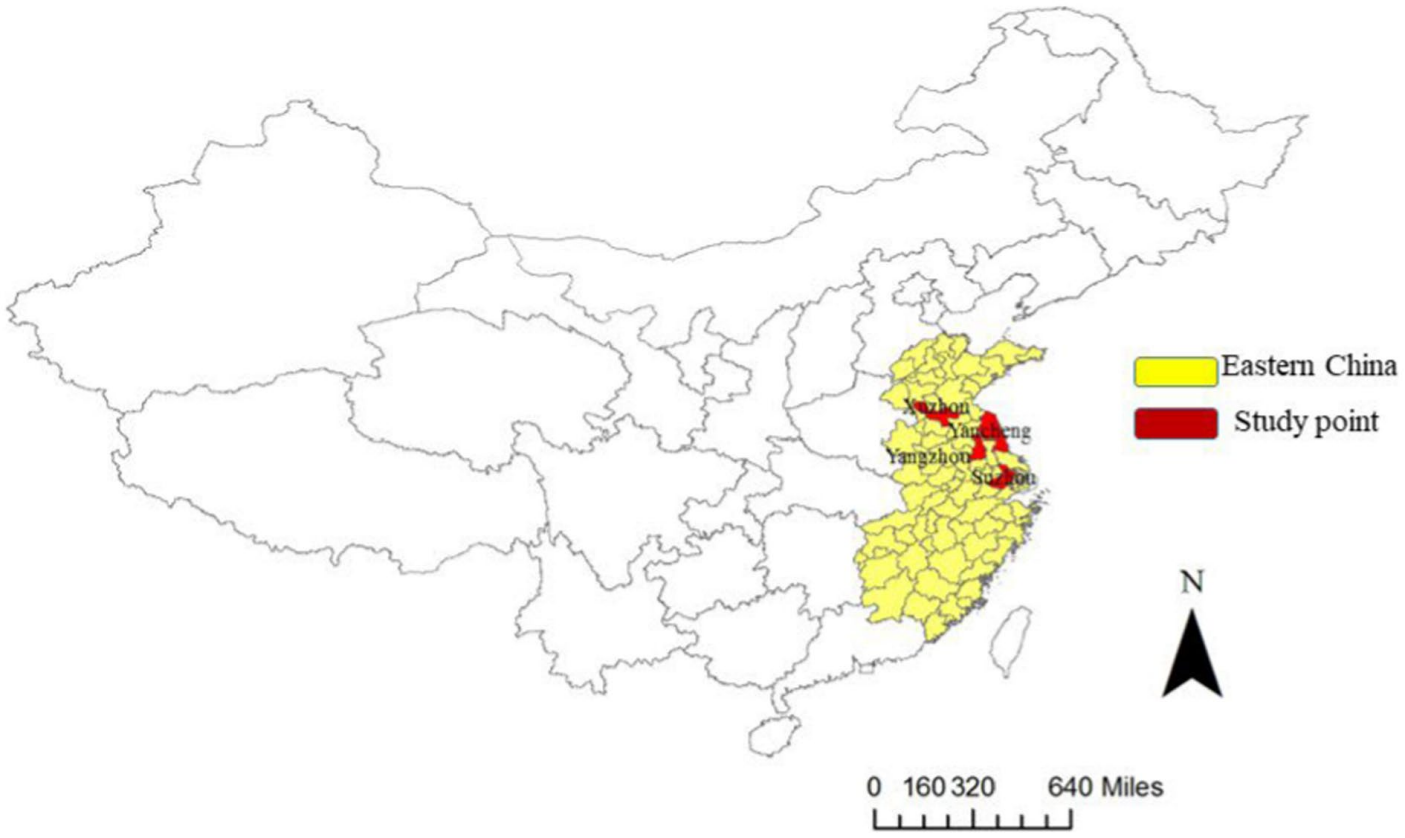
Map of China and the location of the Eastern China Student Health and Wellbeing Cohort Study, including Pei county, Yizheng city, Changshu city, and Dongtai city.

**Figure 2:**
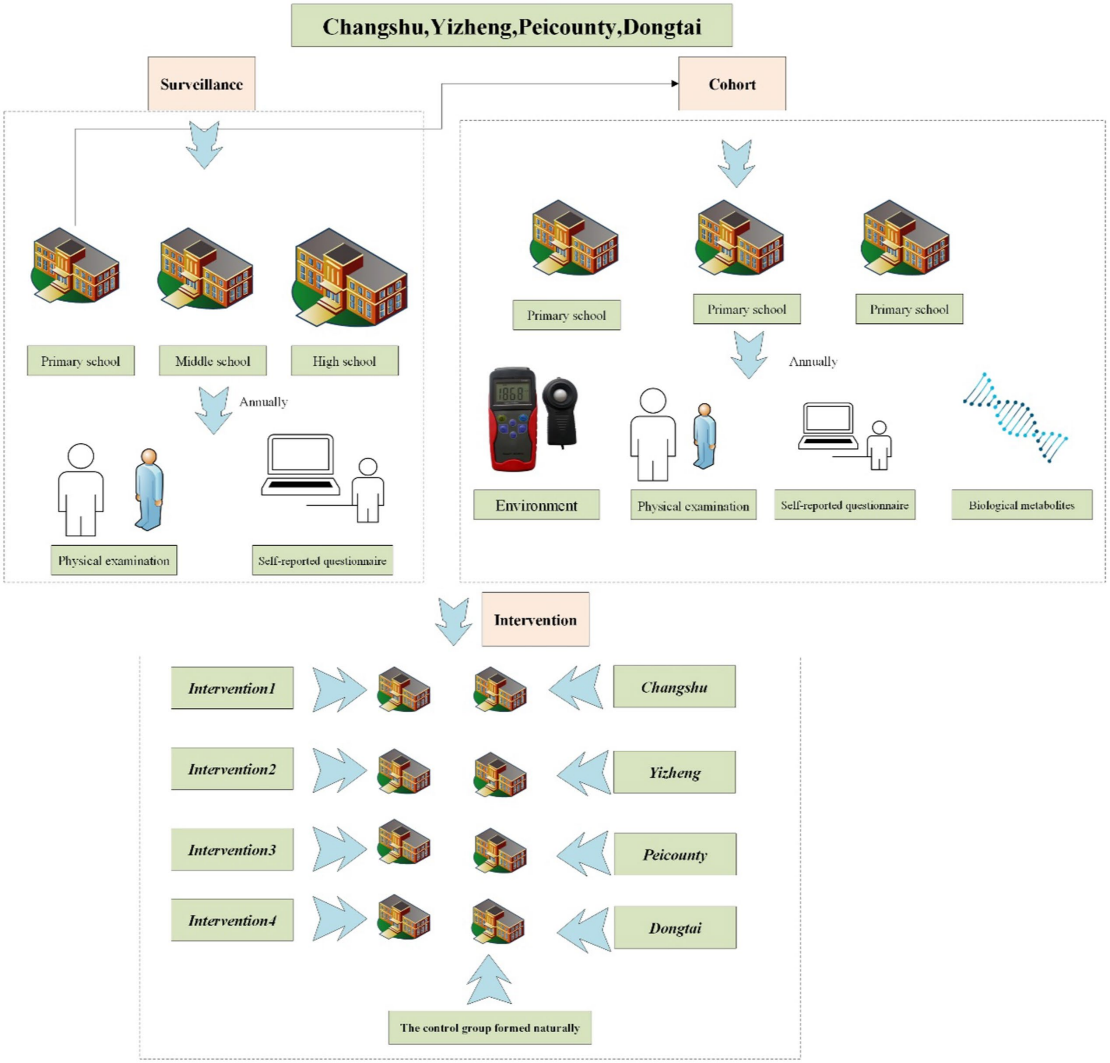
The flowchart of the organization, management, and implementation in the ES-SCI Study (Eastern Chinese Students common disease Surveillance, Cohort, and Intervention study).

We employed a longitudinal design in the **Eastern China Student Health and Wellbeing Cohort Study**, which randomly selected multiple schools from Peicounty, Yizheng, and Changshu. All these municipalities are located in geographically clustered Jiangsu Province of eastern China, which covers approximately 10% of China’s total land area, comprises 30% of China’s population, and contributes to 40% of China’s GDP. Each of these municipalities represents a distinctive level of socioeconomic development, i.e., developed region (34): Public budget revenue>5 billion RMB, such as Changshu (advanced-developed city) and Dongtai (steady-developed city); and developing region: Public budget revenue<5 billion RMB, such as Peicounty (rapid-developing city) and Yizheng (steady-developing city) (Figures 1 and 2).

### How often have they been followed Up?

**Eastern China Student Health and Wellbeing Cohort Study** was launched in 2019 and will be followed up once a year. This is a dynamic cohort study conducted by Jiangsu Provincial Center for Disease Control and Prevention focusing on public health applications. Every 3 years is a stage, and an assessment will be made. The first stage is 2019–2021 and the next stage is 2022–2024. While 9-year-olds may exit the cohort in 3 years when most of them will graduate from primary school, new batches of students will enter the cohort after graduation from pre-school. We collected cohort information every year and integrated it into routine CDC work.

### What Has been measured?

(1) Outcomes-targeted disease:

① Definitions: ***Myopia*** was defined as −0.50 diopters (D) in the worse eye, which was defined as the eye with worse eye (35). ***Significant anisometropia*** was defined as a spherical equivalent, an interocular difference of ≥1.00 D. ***Astigmatism*** was reported as negative cylinder refraction of ≥1.00 DC (36). ***Pre-myopia*** was defined according to a combination of baseline refraction (a refractive state of an eye of 0.75 D and > −0.50 D in children), age, and other quantifiable risk factors demonstrating a sufficient likelihood of the future development of myopia to merit preventative interventions (37). ***Overweight and obesity*** were defined by the *Screening for overweight and obesity among school-age children and adolescents* (WST586-2018) and BMI is specified as weight in kilograms divided by height in meters squared (38). ***Malnutrition*** was defined by the value of BMI, and a specific definition can be referred to *Screening standard for malnutrition of school-age children and adolescents (WS/T 456–2014)* (39). ***Elevated blood pressure*** was defined using the National Blood Pressure Reference for Chinese Han Children and Adolescents (CCBP) thresholds, i.e., ≥95th percentile as elevated BP (40). ***The Center for Epidemiologic Studies Depression (CES-D) Scale*** contains 20 items about symptoms that occurred in the week before the interview with response options from 0 to 3 that refer to the frequency of the symptoms. The score ranges between 0 (best possible) and 60 (worst) and the cutoff point that has been typically recommended for depression is 16 (41).
② Annual physical examination: Doctors in local hospitals are responsible for the physical examination of students. (1) ***Ophthalmic measurement:*** Students have participated in an ophthalmic examination with an auto-refractor (Topcon KR-800) applied with cycloplegia. The cycloplegic refraction is measured using tropicamide phenylephrine eye drops (mydrin) every 5 min, three times. Then, refractive error is measured 30 min after the first drop of tropicamide by autorefractor with five repeated measurements. (2) ***Height, weight, and blood pressure:*** Anthropometric measurements were taken to obtain data on height, weight, and blood pressure according to standard techniques (42, 43). The model of the sphygmomanometer is Omron HBP-1300. ***Mental health questionnaire surveys*:** We have employed validated survey instruments. Individuals with a score of 16 or more must have had either at least 6 of the 20 symptoms in the CES-D with persistence for most of the previous week, or a majority of the symptoms on the scale for shorter periods. CES-D literacy level has been defined as easy, and it takes between 2 and 5 min to complete (44, 45).
(2) Exposures: Individual behaviors, environment and metabolomics, and gene and epigenetics

① ***Questionnaire surveys***: Standardized myopia questionnaire (46) and National Questionnaire on Students’ health status and Influencing factors (47) collect health-related data once a year since baseline in addition to sociodemographic data such as family type, delivery mode, house area, parental education level, and lifestyle data, such as smoking, alcohol consumption, sleep time, exposure to sunlight, exercise, and diet.
② ***Biological sample collection*:** We collect biological samples *per annum* including urine, saliva, and feces from 1,200 participants in the school.
③ ***Environment measurement:*** We have also measured the classroom environment indicators annually including classroom area *per capita* (per m2), blackboard illumination (Lx), blackboard size (m2), blackboard reflectance, uniformity of blackboard, desk illumination (lx), uniformity of class table, and level of noise (dB).
(3) Quality management and data analysis
  Strict quality measures were in place including regular review of all questionnaires to enhance completion and call-back where appropriate to minimize missingness in all data fields by research assistants. Sensitive records are strictly kept confidential with restricted access to non-investigators (Supplementary Figure 1). Data mining in-depth would be conducted in subsequent research, such as crosstab analysis, regression analysis, and mediation analysis.

## Findings to date

### Cohort profile of the eastern China student health and wellbeing cohort study

The first follow-up was conducted in 2019 with 3,257 students from developing regions. The second follow-up was arranged in 2020 with 1 year duration with the original 3,257 students from developing regions as well as 1,643 newly added students from developed regions. The third-year follow-up was arranged in 2021 with the 1-year duration with a total of 6,506 students from the study sites (Peicounty, Yizheng, Changshu, and Dongtai). The ratio of male to female is 1.16 among a total of 6,506 student participants, of which 1,643 (25.3%) students are from Changshu, 1,762 (27.1%) students from Peicounty, 966 (14.8%) students from Yizheng, and 2,135 (32.8%) students from Dongtai. There are 464 (7.1%) 6-year-olds, 1,169 (18.0%) 7-year-olds, 1,461 (22.5%) 8-year-olds, 1,675 (25.7%) 9-year-olds, 1,120 (17.2%) 10-year-olds, 599 (9.2%) 11-year-olds, and 18 (0.3%) 12-year-olds (Table 1).

**Table 1:** Cohort profiles of students in the Eastern China Student Health and Wellbeing Cohort Study.

	2019 year No. (%)	2020 year No. (%)	2021 year No. (%)
**District** ^**a**^
Developed region	–	1,643 (33.5)	2,728 (41.9)
Developing region	3,257 (100.0)	3,257 (66.5)	3,778 (58.1)
**Gender**
Male	1,768 (54.3)	2,610 (53.3)	3,491 (53.7)
Female	1,489 (45.7)	2,290 (46.7)	3,015 (46.3)
**Age**
6~	397 (12.2)	413 (8.4)	464 (7.1)
7~	1,100 (33.8)	875 (17.9)	1,169 (18.0)
8~	1,094 (33.6)	1,540 (31.4)	1,461 (22.5)
9~	644 (19.8)	1,342 (27.4)	1,675 (25.7)
10~	22 (0.6)	708 (14.4)	1,120 (17.2)
11~	-	22 (0.5)	599 (9.2)
12~	-	-	18 (0.3)
Total	3,257 (100.0)	4,900 (100.0)	6,506 (100.0)

a Developed region: Public budget revenue>5 billion RMB; Developing region: Public budget revenue<5billion RMB.

Results are summarized as annual physical examination, questionnaire surveys, bio-sampling completion status, and environmental measurements

(1) *Annual physical examination:* Of all the cohort participants, the prevalence of myopia, obesity, and elevated blood pressure is 21.8%–29.2%, 17.4%–29.7%, and 12.6%–32.5% in the first year, respectively, and 48.4%–50.4%, 3.0%–5.0%, and 4.2%–18.3% in the third year, respectively. The average axial length R/L with standard deviation (SD) is (23.1 ~ 23.3)/(23.1 ~ 23.2) mm in the first year and 23.8/(23.7 ~ 23.8) mm in the third year. The average spherical equivalent R/L is (0.00–0.35)/(0.09–0.39) D in the first year and (−0.69–0.65)/(−0.35–0.55) D in the third year. The average height is (127.5–132.3) cm in the first year and (139.3–144.0) cm in the third year. The average weight is (28.7–31.4) kg in the first year and (38.5–40.9) kg in the third year (Table 2).
(2) *Questionnaire surveys:* The questionnaire topics include diet (such as the habit of brushing your teeth every day morning and night accounted for 68.7%, Never consumption of sugary drinks accounted for 30.1%), physical exercise (such as high-intensity exercise 0 day per week accounted for 33.7%), bullying (such as parents never criticize children accounting for 90.8%), and family (housing area >200 m^2^ accounting for 11.8%). Parent symptom questionnaire (conners) (48) comprises 48 items and 6 subscales: conduct problems (4.0 ± 3.9), difficulties in learning (2.8 ± 2.1), psychosomatic disorders (0.3 ± 0.8), impulsivity/hyperactivity (2.0 ± 2.0), anxiety (1.1 ± 1.2), and hyperactivity index (4.7 ± 4.0) (Table 3).
(3) *Bio-sampling completion status:* Current progress or plan information of the cohort study concerning biological samples and other related surveys can be referred to in Table 4.
(4) *Environment measurements:* According to the national standards GB 50099–2011, the classroom area *per capita* is greater than and equal to 1.36 m^2^/person. The average desk illumination is 430.78 (355.84–611.56) L_X_ and the average blackboard illumination is 365.33 (286.83–516.84) L_X_. Some details can be found in Table 5.

**Table 2:** Baseline and 2-year follow-up of annual physical examination of Eastern China Student Health and Wellbeing Cohort Study.

		Developed region	Developing region
Myopia, %	Baseline	29.2	22.3
	First-year	46.0	38.3
	Second-year^	-	49.1
Obesity, %	Baseline	17.4	20.7
	First-year	19.1	22.3
	Second-year	-	4.3
Elevated blood pressure, %	Baseline	12.6	17.1
	First-year	12.2	13.8
	Second-year	-	9.2
SE R/L, D	Baseline	0.20 ± 1.36/0.28 ± 1.43	0.31 ± 1.45/0.38 ± 1.40
	First-year	−0.33 ± 1.55/−0.30 ± 1.59	−0.24 ± 1.56/−0.11 ± 1.56
	Second-year	-	−0.67 ± 1.72/−0.48 ± 2.42
Axial length R/L, mm	Baseline	23.3 ± 0.9/23.2 ± 0.9	23.2 ± 0.9/23.2 ± 0.9
	First-year	23.6 ± 0.9/23.5 ± 1.4	23.6 ± 0.9/23.5 ± 0.9
	Second-year	-	23.8 ± 1.0/23.8 ± 1.1
Corneal curvature R/L	Baseline	43.1 ± 1.4/43.1 ± 1.4	42.7 ± 1.4/43.0 ± 1.6
	First-year	42.9 ± 2.0/42.8 ± 1.9	43.0 ± 1.4/43.0 ± 1.4
	Second-year	-	43.0 ± 1.5/43.0 ± 1.5
BMI kg/m^2^	Baseline	17.2 ± 3.6	17.4 ± 3.6
	First-year	18.5 ± 6.4	18.4 ± 3.8
	Second-year	-	19.5 ± 4.4
Weight, kg	Baseline	28.7 ± 7.5	30.4 ± 8.3
	First-year	32.9 ± 9.9	34.4 ± 10.1
	Second-year	-	40.1 ± 11.8
Height, cm	Baseline	128.5 ± 8.1	131.3 ± 7.5
	First-year	133.4 ± 9.1	135.2 ± 15.2
	Second-year	-	142.3 ± 8.8
SBP, mmHg	Baseline	98.9 ± 10.3	106.4 ± 10.0
	First-year	101.8 ± 11.0	106.4 ± 10.3
	Second-year	-	106.6 ± 10.0
DBP, mmHg	Baseline	65.6 ± 8.5	62.6 ± 8.0
	First-year	63.8 ± 7.8	64.1 ± 7.7
	Second-year	-	63.0 ± 7.4

**Table 3:** Characteristics of school-based student health questionnaire surveys.

	Source of data	Proportion(%) or Mean ± SD			
**Basic information**		Developed region (3,778) + Developing region (3,257)			
Child’s nationality	Parents	Han (99.2)			
Numbers of family members living with your children in the past half year	Parents	4.03 ± 1.40			
Father education?	Parents	University (37.0)	Highschool (33.6)	Middle school (26.0)	
Mother education?	Parents	University (34.8)	Highschool (30.6)	Middle school (30.8)	
Breastfed at birth?	Parents	Yes (90.3)			
How long breastfed?	Parents	>1 year (20.0)	7–9 months (20.7)	1–3 months (6.7)	
Family income?	Parents	>10,000 RMB/Mon (28.0)		<2,000 RMB/Mon (3.6)	
Area of your family housing	Parents	200 M^2^ (11.8)	150 M^2^ (9.7)	100 M^2^ (41.7)	
**Diet and physical activity**
The habit of brushing teeth	Parents	Morning and evening (68.7)	Morning (25.0)	Evening (4.5)	Once in a while (1.8)
During the past 7 days, how many times did you drink sugary drinks?	Parents	Never (30.1)	Less than one per day (68.2)	More than one time per day (1.7)	
During the past 7 days, how many times did you eat sugary food?	Parents	Never (7.6)	Less than one time per day (84.9)	More than one time per day (7.5)	
During the past 7 days, how many times did you eat fried food	Parents	Never (9.9)	Less than one time per day (87.8)	More than one time per day (2.3)	
During the past 7 days, how many times did you eat fresh fruits?	Parents	Never (1.9)	Less than one time per day (31.7)	One time per day (58.4)	More than 2 times per day (8.0)
During the past 7 days, how many times did you eat fresh vegetables?	Parents	Never (5.6)	Less than one time per day (23.3)	One time per day (49.2)	More than 2 times per day (21.9)
Does your child eat breakfast every day?	Parents	Often (95.8)	Once in a while (4.0)	Never (0.2)	
How many days can your child do moderate and high-intensity exercise every day (at least 60 min or more in total)?	Parents	0 day (33.7) 1 day (21.1)	2 days (18.3) 3 days (11.9)	4 days (4.1) 5 days (4.7)	6 days (1.6) 7 days (4.6)
How many PE classes does your child have on average every week?	Parents	0 time (0.6) 4 times (18.0)	1 time (14.6) ≥5 times (2.8)	2 times (35.6)	3 times (28.4)
**Bullying related behavior**					
In the past 30 days, has your child been bullied in any of the following forms on or around the campus?					
Be maliciously teased?	Parents	Never (90.8)	Sometimes (8.9)	Often (0.3)	
Property demanded?	Parents	Never (98.6)	Sometimes (1.3)	Often (0.1)	
Be deliberately excluded from collective activities or isolated?	Parents	Never (97.0)	Sometimes (2.9)	Often (0.1)	
Be threatened and intimidated?	Parents	Never (98.0)	Sometimes (1.9)	Often (0.1)	
Be beaten, kicked, pushed, squeezed or locked in the house?	Parents	Never (93.8)	Sometimes (6.0)	Often (0.2)	
Be teased because of physical defects or appearance?	Parents	Never (98.2)	Sometimes (1.7)	Often (0.1)	
Will you beat and scold your children now?	Parents	Never (38.6)	Sometimes (60.2)	Often (1.2)	
**Tobacco and alcohol use**
Has your child ever smoked, even if only once or twice?	Parents	Yes (0.7)			
Have you seen anyone smoking on the following occasions in the past 7 days?	Parents	Never (22.8)	Home (28.8)	School (1.3)	Other public places (61.2)
Has your child ever drunk a whole glass of wine?	Parents	Yes (0.4)			
Does anyone smoke at home?	Parents	No (38.2)	Father (50.4)	Mother (0.2)	
**Schooling related issues**
Does your child attend indoor extracurricular classes every week? (such as English, piano, etc.)?	Parents	Yes (43.5)			
Does your child attend outdoor extracurricular classes every week? (such as football, basketball and other classes)	Parents	Yes (17.0)			
From Monday to Friday, does your child study or read books, including homework, after school?	Parents	Yes (94.2)			
From Monday to Friday, does your child watch TV after school?	Parents	Yes (30.9)			
From Monday to Friday, does your child use a computer or play games after school?	Parents	Yes (11.5)			
On weekends, in addition to classes, does your child study or read books, including homework?	Parents	Yes (85.9)			
Does your child watch TV on weekends?	Parents	Yes (77.8)			
Do your children use computers or play games on weekends?	Parents	Yes (29.0)			
**Life schedule questionnaire**
What time does your child get up in the morning from Monday to Friday?	Parents	Before 6:00 (5.2)	6:00–7:00 (78.5)	7:00–8:00 (15.6)	
What time does your child get up in the morning on weekends?	Parents	Before 6:00 (1.4)	6:00–7:00 (12.7)	7:00–8:00 (43.3)	
What time does your child go to bed from Monday to Friday?	Parents	Before 21:00 (50.7)	21:00–22:00 (45.2)	22:00–23:00 (3.2)	
What time does your child go to bed on weekends?	Parents	Before 21:00 (30.3)	21:00–22:00 (57.7)	22:00–23:00 (10.8)	
Do your children have outdoor activities during lunch break from Monday to Friday? How long?	Parents	Yes (36.5)	Less than 30 min (93.0)	30-60 min (5.8)	More than 60 min (1.2)
Do your children go to outdoor activities (except outdoor classes) on weekends? How long?	Parents	Yes (83.2)	Less than 30 min (9.5)	30-60 min (40.2)	More than 60 min (50.3)
**Reading and writing habit**
When your child is reading and writing, his chest is more than a punch from the edge of the table?	Parents	Never (12.9)	Once in a while (51.5)	Often (25.0)	Always (10.6)
When reading and writing, your child’s eyes are more than one foot away from the book?	Parents	Never (12.1)	Once in a while (48.9)	Often (28.6)	Always (10.4)
When reading and writing, your child’s fingers are about an inch (3.3 cm) away from the tip of the pen?	Parents	Never (12.1)	Once in a while (37.6)	Often (32.4)	Always (17.9)
Does your child’s teacher remind you that your reading and writing posture is incorrect?	Parents	Never (14.9)	Once in a while (28.4)	Often (37.7)	Always (19.0)
Do you remind your child that his / her reading and writing posture is incorrect?	Parents	Never (5.0)	Once in a while (19.4)	Often (48.3)	Always (27.3)
**Screen usage**
In the past week, how long did your child watch TV (including video games such as X-box) on average every day?	Parents	Never (24.2) 1 h (50.9)	1-2 h (18.6) 2-3 h (4.2)	3-4 h (1.1) 4 h or more (1.0)	
In the past week, how much time does your child usually use the computer every day?	Parents	Never (69.0) 1 h (24.2)	1-2 h (5.5) 2-3 h (0.9)	3-4 h (0.2) 4 h or more (0.2)	
How long has your child used mobile electronic devices (including mobile phones, handheld game consoles, tablets, etc.) in the past week?	Parents	Never (52.0)		1.03 ± 0.99 h	
Does your child read books or use electronic screens in direct sunlight?	Parents	Never (71.2)	Once in a while (27.1)	Often (1.3)	Always (0.4)
Does your child turn off the light when looking at the electronic screen after dark?	Parents	Never (74.5)	Once in a while (21.7)	Often (2.7)	Always (1.1)
Will your child read books or electronic screens lying or lying on his stomach?	Parents	Never (41.4)	Once in a while (50.1)	Often (7.8)	Always (0.7)
Does your child read books or use electronic screens when walking or riding?	Parents	Never (73.9)	Once in a while (24.1)	Often (1.7)	Always (0.3)
**Family environment questionnaire (FES-CV) (except for Dongtai)**
Intimacy	Parents	8.2 ± 1.3			
Contradiction	Parents	2.0 ± 1.6			
Success	Parents	5.0 ± 1.9			
Intellectual	Parents	4.8 ± 1.9			
Entertainment	Parents	4.4 ± 2.2			
Organization	Parents	6.7 ± 1.5			
Controllablity	Parents	5.0 ± 2.2			
**Parent Symptom Questionnaire(conners) (except for Dongtai)**
Conduct problems	Parents	4.0 ± 3.9			
Difficulties in learning	Parents	2.8 ± 2.1			
Psychosomatic disorders	Parents	0.3 ± 0.8			
Impulsivity/hyperactivity	Parents	2.0 ± 2.0			
Anxiety	Parents	1.1 ± 1.2			
hyperactivity	Parents	4.7 ± 4.0			
CES-D scores	Students	12.9 ± 9.8/11.6 ± 9.0			
Other related survey					
Questionnaires (Internet addiction questionnaire, Sleep status questionnaire, Academic performance, Parental related behaviors, psychological scale survey)	All students	Un-finished			

**Table 4:** Bio-sampling completion status (exposures).

	Sample size	Collection year	Source of samples
Saliva			
SNP (rs524952, rs524952, rs2969180, rs2908972, rs10880855, rs1939008, rs9928731, rs72621438, rs9939609, rs8050136 and so on)	1,200	2021–2022 academic year	Student
Urine			
Metabolites (MMP, MEP, MBP, MBzP, MEHP, NP, BPA, DES, 4-OP, BPB, PB, EP, MP, EE2, DES and so on) Bisphenol A(0.734 ng/ml)	1,200	2021–2022 academic year	Student
Faeces			
Metabolites (Eubacterium, Coprococcus, Sanguibacteroide, Pseudoramibacte, Plesiomonas, Acetobacterium, Prevotella, Sarcina, Peptoclostridium, Kosakonia, and so on)	1,200	2022–2023 academic year	Student

**Table 5:** Environment-related indicators measurements.

	Source of data	Proportion (%) or Mean ± SD		Source of data	Proportion (%) or Mean ± SD
Class environment	Parents				
*Per capita* classroom area	CDC investigation	1.14 ± 0.45	25.3	≥1.36 m^2^/person (GB 50099–2011)	
Compliance of desks and chairs	CDC investigation	0.01 ± 0.05	0.0	≥80% (GB/T3976-2014)	
Blackboard size	CDC investigation	4.84 ± 0.14	100.0	3.6 m × 1.0 m (GB 28231–2011)	
Blackboard average reflectance	CDC investigation	0.14 ± 0.03	60.8	0.15 ~ 0.20 (GB 7793–2010)	
Average illumination of blackboard surface	CDC investigation	365.33 (286.83–516.84)	27.8	≥500 lx (GB 7793–2010)	
Blackboard evenness	CDC investigation	0.79 ± 0.16	60.8	≥0.8 (GB 7793–2010)	
The average desk illumination	CDC investigation	430.78 (355.84–611.56)	87.3	≥300 lx (GB 7793–2010)	
Class table evenness	CDC investigation	0.52 ± 0.18	20.3	≥0.7 (GB 7793–2010)	
Wearable devices	CDC investigation	Un-finished			
Air environment surveillance	CDC investigation	Un-finished			

## Discussion

**The Eastern China Student Health and Wellbeing Cohort Study** is aiming to focus on student common diseases. Health outcomes in relation to influencing factors will be quantified during the follow-up period. This is a dynamic cohort study conducted by Jiangsu Provincial Center for Disease Control and Prevention focusing on public health applications. Every 3 years is a stage, and an assessment will be made. For children with targeted diseases, we will continuously observe the relationship between the related indicators of common diseases and exposure factors in students in the future, e.g., for myopes, to quantify associations between myopic-related indicators such as axial length or spherical equivalent values and individual behaviors. For children without targeted diseases, we will explore the mechanism and onset of diseases. A longitudinal study design looking into the onset or development of targeted diseases in children and adolescents may identify potential interventional strategies to reduce the disease burden of relevance (49, 50). This unique platform generates evidence for policy and practice translation to improve health and wellbeing in school-aged children and adolescents.

**The Eastern China Student Health and Wellbeing Cohort Study** has the following strengths. First, the establishment of this student cohort in one of the most affluent regions in China. Study findings will provide evidence to the public and authorities of targeted disease burden among children and its impact on school-aged child health. Second, the employment of a classic epidemiological study design using unified diagnostic criteria to measure obesity, elevated blood pressure, myopia, and mental health will provide the best available estimate of the disease burden in school-aged children and adolescents. Third, with the comprehensive use of public health and clinical indicators, the study would further establish early warning models of common diseases in students. Fourth, the innovative epigenetic analysis will be carried out to complement what we have done previously in regard to joint effects of gene–environment on the development of targeted disease (51, 52). Fifth, the provision of a robust longitudinal cohort platform with breadth and depth of data for innovative research as well as evidence translation into health policies and practices to improve health and service in school-aged children. Sixth, the eastern student cohort study tends to provide evidence for intervention studies.

## Data information

All relevant data are shown within the manuscript, but original datasets cannot be shared because of students’ privacy. However, possible collaborators are invited to contact the ES-SCI work group primary investigator Xiyan Zhang (xyzhang0220@foxmail.com).

## Ethics statement

The studies involving human participants were reviewed and approved by Institutional Review Board of Ethics committee of Jiangsu Provincial Center for Disease Control and Prevention. Written informed consent to participate in this study was provided by the participants’ legal guardian/next of kin.

## Author contributions

XZ, WY, and YZ: data curation. YX and XW: investigation. YW: methodology. FZ and WD: project administration. GZ, FT, JM, YT, and CP: design and advice. WD and YW: article modification. YZ, JY, and WD: supervision. XZ, WY, FZ, and WD: writing the original draft. All authors contributed to the article and approved the submitted version.

## Funding

Annually basic public health of the child and adolescent health (student common disease intervention) funding was covered by the Jiangsu Provincial Department of Finance and Jiangsu Provincial Health Committee for 2019–2020 [2019 64], 2020–2021 [2020 124] [2020 52], 2021–2026 (un-issued), respectively. The role of the funder(s) is not applicable in this study. This research was supported by a demonstration project of comprehensive prevention and control of emerging infectious diseases (BE2015714), http://www.jshealth.com/xxgk/sewcjz/xmzl/sbjkt/201708/t20170830_59379.html (FZ).

## Conflict of interest

The authors declare that the research was conducted in the absence of any commercial or financial relationships that could be construed as a potential conflict of interest.

## Publisher’s note

All claims expressed in this article are solely those of the authors and do not necessarily represent those of their affiliated organizations, or those of the publisher, the editors and the reviewers. Any product that may be evaluated in this article, or claim that may be made by its manufacturer, is not guaranteed or endorsed by the publisher.

## ES-SCI work group (Eastern Chinese Students common disease Surveillance, Cohort and Intervention study)

Hui Shen, Jia Hu, Suzhou Center for Disease Control and Prevention; Dongyue Wang, Mingzhu Shen, Yingni Sun, ChangsShu Center for Disease Control and Prevention; Yueqian Zhu,Weiwei Zhu, Yangzhou Center for Disease Control and Prevention; Zilin Li, Limin Huang, Yindi Xu, Shaochun Chen, Zaiping Mao, Yizheng Center for Disease Control and Prevention; Jimin Xu, Shenghao Miao, Juan Zhang, Xuzhou Center for Disease Control and Prevention; Wei Wang, Qiang Liu, Ping Li, Qiankun Zhang, Ping Feng, Ying Wang, Qingting Bian, Juan Lu, Peicounty Center for Disease Control and Prevention; Zhongyou Sun, Xiaohong Xu, Zhongyou Sun, Xiaohong Xu; Xiaomeng Huang, Kang Wu, Dongtai Center for Disease Control and Prevention; Sunan Chen, Yiwen Hua, Feng Huang, Southeast University.
