# Supplementary figures and images for: Design, methodology, and baseline of eastern China student health and wellbeing cohort study
Source: Front Public Health. 2023 Apr 27;11:1100227. doi: 10.3389/fpubh.2023.1100227 (PMC10173362; doi:10.3389/fpubh.2023.1100227)

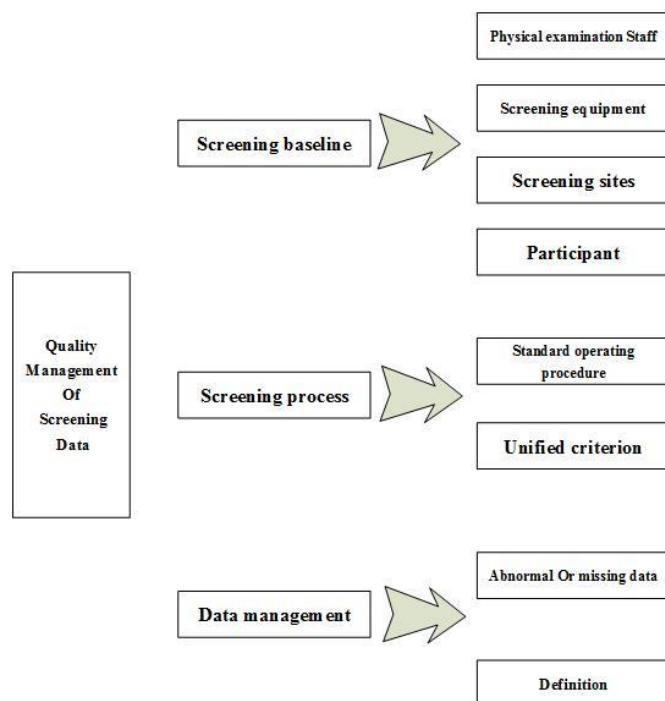

Supplement Figure1 Quality management of myopia screening data of ES-SCI Study

Supplement: Supplementary file 1 [file Image_1.pdf]
